# Specificity Determinants for Lysine Incorporation in *Staphylococcus aureus* Peptidoglycan as Revealed by the Structure of a MurE Enzyme Ternary Complex*

**DOI:** 10.1074/jbc.M113.508135

**Published:** 2013-09-24

**Authors:** Karen M. Ruane, Adrian J. Lloyd, Vilmos Fülöp, Christopher G. Dowson, Hélène Barreteau, Audrey Boniface, Sébastien Dementin, Didier Blanot, Dominique Mengin-Lecreulx, Stanislav Gobec, Andréa Dessen, David I. Roper

**Affiliations:** From the ‡ School of Life Sciences, Gibbet Hill Road, University of Warwick, Coventry CV4 7AL, United Kingdom,; the § University Paris-Sud, Laboratoire des Enveloppes Bactériennes et Antibiotiques, Institut de Biochimie et Biophysique Moléculaire et Cellulaire, Unité Mixte de Recherche (UMR) 8619 CNRS, F-91405 Orsay, France,; the ¶ Faculty of Pharmacy, Aškerčeva 7, University of Ljubljana, 1000 Ljubljana, Slovenia, and; the ‖ Bacterial Pathogenesis Group, Institut de Biologie Structurale, Université Grenoble I, Centre National de la Recherche Scientifique, Commissariat à l'Enérgie Atomique, 41 rue Jules Horowitz, 38027 Grenoble, France

**Keywords:** Amino Acid, Antibiotic Resistance, Bacterial Metabolism, Peptidoglycan, X-ray Crystallography, MurE, Enzyme Kinetics, Structural Biology

## Abstract

Formation of the peptidoglycan stem pentapeptide requires the insertion of both l and d amino acids by the ATP-dependent ligase enzymes MurC, -D, -E, and -F. The stereochemical control of the third position amino acid in the pentapeptide is crucial to maintain the fidelity of later biosynthetic steps contributing to cell morphology, antibiotic resistance, and pathogenesis. Here we determined the x-ray crystal structure of *Staphylococcus aureus* MurE UDP-*N*-acetylmuramoyl-l-alanyl-d-glutamate:meso-2,6-diaminopimelate ligase (MurE) (E.C. 6.3.2.7) at 1.8 Å resolution in the presence of ADP and the reaction product, UDP-MurNAc-l-Ala-γ-d-Glu-l-Lys. This structure provides for the first time a molecular understanding of how this Gram-positive enzyme discriminates between l-lysine and d,l-diaminopimelic acid, the predominant amino acid that replaces l-lysine in Gram-negative peptidoglycan. Despite the presence of a consensus sequence previously implicated in the selection of the third position residue in the stem pentapeptide in *S. aureus* MurE, the structure shows that only part of this sequence is involved in the selection of l-lysine. Instead, other parts of the protein contribute substrate-selecting residues, resulting in a lysine-binding pocket based on charge characteristics. Despite the absolute specificity for l-lysine, *S. aureus* MurE binds this substrate relatively poorly. *In vivo* analysis and metabolomic data reveal that this is compensated for by high cytoplasmic l-lysine concentrations. Therefore, both metabolic and structural constraints maintain the structural integrity of the staphylococcal peptidoglycan. This study provides a novel focus for *S. aureus*-directed antimicrobials based on dual targeting of essential amino acid biogenesis and its linkage to cell wall assembly.

## Teaser

**Background:** MurE controls stereochemical incorporation of lysine or diaminopimelate into peptidoglycan stem peptides.

**Results:** The structure of *S. aureus* MurE reveals an unexpected lack of specificity for lysine within the active site.

**Conclusion:** Incorporation of lysine is supported by the comparatively high concentration of cytoplasmic lysine, not enzyme specificity.

**Significance:** This study provides new perspectives in targeting Gram-positive peptidoglycan assembly for antimicrobial discovery.

## Introduction

Peptidoglycan (PG)^4^ is an essential cell wall component of almost all bacteria (an exception being the wall-less Mycoplasmas) (1). This fact, together with its uniqueness to only the (eu)bacterial cell, has made the enzymatic machinery responsible for the biosynthesis of PG a prime target for antimicrobial therapy. Ever since the discovery and use of penicillin in the early 1940s, penicillin and other antibiotics that target PG in bacterial cell wall biosynthesis have been cornerstones in our fight against infection.

The PG synthesis pathway offers a wide range of valid intracellular and extracellular targets for drug discovery. The biosynthesis of PG occurs in three stages, each involving separate compartments within the bacterial cell (reviewed in Refs. 2–4). The PG precursor pathway is initiated in the cytoplasm, wherein UDP-*N*-acetylglucosamine is acylated with phosphoenol pyruvate in a reaction catalyzed by MurA (5). The resulting enol-pyruvoyl-UDP-*N*-acetylglucosamine is reduced with NADPH by MurB, yielding UDP-*N*-acetylmuramic acid (UDP-MurNAc). This intermediate is then subjected to ATP-dependent aminoacylation with, in sequence, l-alanine, d-glutamate, and either l-lysine or *meso*-diaminopimelic acid (mDAP), by the sequential action of Mur ligases C, D, and E to produce UDP-MurNAc-tripeptide. The final cytoplasmic PG precursor, UDP-MurNAc-pentapeptide, is generated by MurF, which, in the presence of ATP, appends d-alanyl-d-alanine onto the carboxyl terminus of the UDP-MurNAc-tripeptide. The d-alanyl-d-alanine dipeptide is generated from d-alanine by d-alanine:d-alanine ligase (Ddl), again in an ATP-dependent aminoacylation.

The phospho-MurNAc-pentapeptide moiety is then transferred to the membrane stage of synthesis by coupling to C_55_ undecaprenyl-phosphate via the integral membrane protein MraY to form lipid I (6–8). Lipid I is then glycosaminylated by UDP-GlcNAc to yield lipid II via the action of MurG (9, 10). At this point, the pentapeptide stem of lipid II can be further modified by, for example, aminoacylation of the ϵ-amino group of the third amino acid residue on the pentapeptide stem (see below) or amidation of glutamate at position 2 to iso-glutamine (11, 12) depending on the species. Finally, the lipid precursor is translocated through the cytoplasmic membrane (13), to be polymerized by a battery of transglycosylase enzymes (14). In this stage occurring on the extracellular face of the cytoplasmic membrane, the pentapeptides present in the nascent PG strands are cross-linked by transpeptidation in a series of reactions catalyzed by penicillin-binding proteins (PBPs) (15). Specifically, these enzymes cross-link the ϵ-amino group of the l-lysine or mDAP residue added by MurE to adjacent stem peptides.

The insertion of the third position amino acid into the stem peptide in the cytoplasmic phase of PG biosynthesis is a pivotal point in the biosynthetic pathway. It clearly has a profound implication for later PBP-dependent steps in biosynthesis. The overexpression of the *Staphylococcus aureus murE* gene (*murE*_Sa_) in *Escherichia coli* has been shown to be lethal (16) as the extracellular *E. coli* PBP transpeptidases are unable to utilize a l-lysine-containing PG precursor. In addition, recent studies of MurE from the thermophile, *Thermotoga maritima*, show that incorporation of d-lysine produces unusual peptidoglycan intermediates that are involved in peptidoglycan cross-linking patterns (17, 18). The overwhelming substrate specificity of the MurE enzymes in *E. coli* and *S. aureus* for mDAP and l-lysine, respectively (19), is consistent with the key role of MurE by ensuring that the correct third position amino acid is inserted into the pentapeptide stem of PG with respect to further maturation of the PG cell wall by high molecular weight transpeptidases. Additionally, in *S. aureus*, a series of glycyl-tRNA^Gly^-dependent Fem ligases (FemX, -A, -B) (20) are required to add a pentaglycine side chain to the ϵ-amino group of the l-lysine inserted in the pentapeptide stem by MurE. These enzymatic steps produce a lipid-II-(Gly_5_) precursor, which is essential for growth, maturation, and embellishment of *S. aureus* PG because deletions of these genes either are lethal or impair growth, leading to aberrant septum formation and lowered methicillin resistance levels (20). Furthermore, in other Gram-positive pathogens with branched stem peptides, such as *Streptococcus pneumoniae,* replacement of the third position l-lysine with mDAP of the stem peptide completely eliminates the ability of the Fem homologue MurM to aminoacylate the lipid II precursor (21). The third position amino acid therefore constitutes an important center for elaboration of the stem peptide of PG with further amino acids, which provide the anchoring point for a variety of cell wall biomolecules via the action of sortase enzymes that are essential for virulence and pathogenesis (22). The substrate and product of the MurE reaction are also central components in the eukaryotic innate immunity signaling mechanisms mediated by NOD1 and NOD2 receptors allowing detection and response to Gram-negative and Gram-positive bacteria, respectively (15, 23–26).

Thus far, the literature contains only structural descriptions of mDAP dependent MurE enzymes (27, 28) and the d-lysine utilizing MurE of *T. maritima* in an ADP-bound form only (18, 29). Here, we have structurally characterized for the first time a lysine-dependent MurE from *S. aureus* (MurE_Sa_) bound to its UDP-MurNAc tripeptide product as well as in a ternary complex with product and ADP. These high-resolution structures have allowed us to understand the process of discrimination between l-lysine and m-DAP incorporation into the third position of the stem peptide in atomic detail. Mutagenesis of residues involved in l-lysine binding and *in vivo* studies of mutant MurE strains reveal that *S. aureus* MurE has a limited capacity to select its substrate, but the chemical integrity of the peptidoglycan is ensured by elevated levels of l-lysine in the cytoplasm. This work provides new insight into mechanistic details of this essential enzyme, a potential target for the development of new antibacterial agents and the metabolism of lysine in *S. aureus*.

## EXPERIMENTAL PROCEDURES

### 

#### 

##### Protein Expression and Crystallization

An expression construct for the production of recombinant MurE_Sa_ was used to produce milligram quantities of the protein for crystallization studies with the following modifications to the published protocol (19). In brief, *E. coli* BL21(DE3):pLysS transformed with plasmids pREP4groESL and pET2160:*murE*_Sa_ was grown in 2YT medium under ampicillin, chloramphenicol, and kanamycin selection at 37 °C until late log phase prior to the addition of isopropyl β-d-1-thiogalactopyranoside to 0.1 mm and continued overnight growth at 22 °C. Tight regulation of the MurE_Sa_ expression system with T7 lysozyme provided by the pLysS system reduces the toxicity previously shown to be associated with expression of MurE_Sa_ (16). The cells were harvested by centrifugation, and a crude extract was obtained by sonication on ice in 25 mm Hepes, 500 mm NaCl, pH 7.5, containing 20 mm imidazole, 0.2 mm PMSF, and 1 μm each of pepstatin and leupeptin (buffer A). Following clarification of the crude extract by centrifugation at 50,000 × *g* at 4 °C for 30 min, MurE_Sa_ was purified by immobilized metal ion chromatography at room temperature on a 5-ml HisTrap-HP column (GE Life Sciences) equilibrated and washed with buffer A containing 50 mm imidazole. Fractions containing MurE_Sa_ were eluted from the column using reverse-direction flow using buffer A with 500 mm imidazole. The peak fraction from this separation was immediately applied at room temperature to a Superdex 200 26/70 size-exclusion column equilibrated in 25 mm Hepes, 200 mm NaCl, and 1 mm DTT, pH 7.5. Further purification of MurE_Sa_ following desalting into 25 mm Hepes, 0.5 mm EDTA, and 0.5 mm DTT, pH 7.5 (buffer B), was achieved by ion exchange chromatography at room temperature using a MonoQ HR5/5 column equilibrated in buffer B and developed in this buffer over a 15-ml gradient between 0 m and 1 m NaCl. Mutants of MurE were made using the QuikChange methodology.

We found that access to freshly prepared protein samples was crucial to obtaining diffraction quality crystals; protein preparations older than 1 week were prone to proteolysis between the domains of the protein, leading to heterogeneous protein samples and twinned crystals. Initial crystallization screens were performed with protein samples either in the presence of (relative to Mur_Sa_ concentration) a 10-fold molar excess of AMP-PNP and UDP-MurNAc-l-Ala-γ-d-Glu (substrate complex) or in the presence of a 10-fold molar excess of ADP and UDP-MurNAc-l-Ala-γ-d-Glu-l-Lys (product complex). The production of UDP-MurNAc-l-Ala-γ-d-Glu and UDP-MurNAc-l-Ala-γ-d-Glu-l-Lys has been previously described (21, 31, 32). Although crystals were formed in three of the conditions, those formed in the substrate complex conditions were not suitable for x-ray diffraction studies. Multiple crystallization conditions were observed for the product complex and were subjected to diffraction studies. In this case, only crystals from Molecular Dimensions Morpheus^TM^ screen (33) condition D9 (0.1 m Tris/Bicine, pH 8.5, 30% (w/v) PEG550MME/PEG20K mix, 0.12 m 1,6-hexanediol, 1-butanol, 1,2-propanediol (racemic), 2-propanol, 1,4-butanediol, 1,3-proanediol mix) were suitable for data collection. For the product, diffraction quality crystals were observed in the Molecular Dimensions Morpheus^TM^ screen condition C5 (0.1 m Na-Hepes/MOPS, pH 7.5, 0.09 m nitrate phosphate sulfate mix, 30% (w/v) PEG550MME/PEG20K mix). Crystals obtained from the screen were directly flash-cooled in liquid nitrogen and stored for data collection.

##### X-ray Data Collection, Structure Determination, and Refinement

X-ray data on the ADP and UDP-MurNAc-l-Ala-γ-d-Glu-l-Lys liganded crystals were collected on the IO2 beamline at the Diamond Light Source synchrotron (Didcot, UK) using an ADSC Q315 CCD detector. All data were indexed, integrated, and scaled using the XDS package (34). Subsequent data handling was carried out using the CCP4 software package (39). Molecular replacement was carried out using the coordinates of *E. coli* MurE (MurE_Ec_; Protein Data Bank (PDB) code 1E8C (28)) as a search model with the PHASER program (35). Refinement of the structure was carried out by alternate cycles of REFMAC (36) and manual rebuilding in O (37). Water molecules were added to the atomic model automatically by Arp/wARP (37).

Data on the crystal liganded with UDP-MurNAc-l-Ala-γ-d-Glu-l-Lys only were collected using a Xenocs GeniX^3D^ Cu HF (High Flux) microbeam x-ray generator with a Mar345 imaging plate. The data were processed with iMOSFLM (38) and reduced with SCALA from the CCP4 suite (39). The MurE_Sa_·UDP-MurNAc-l-Ala-γ-d-Glu-l-Lys complex was solved by molecular replacement using MolRep (40) and the protein atomic coordinates of the product complex. The structure was refined using iterative cycles of REFMAC (36) and model building/solvent addition with COOT (41). Translation/libration/screw motion, as determined by the TLSMD web server (42), was included in refinement. For both structures, the space group was C2, and there was one molecule in the asymmetric unit. A summary of the data collection and refinement statistics is given in Table 1. Figures were drawn using PyMOL (43).

**TABLE 1:** **Summary of crystallographic data collection and refinement statistics** Numbers in parentheses refer to values in the highest resolution shell.

	MurE_Sa_ and UDP-MurNAc-Ala-Glu-Lys	MurE_Sa_, UDP-MurNAc-Ala-Glu-Lys, and ADP
**Data collection**		
Synchrotron radiation/home source, detector and wavelength (Å)	Xenocs GeniX^3D^ Cu HF, Mar345 detector, 1.5418	Diamond, IO2, ADSC Q315 CCD 0.9795
Unit cell (a, b, c (Å), β (°))	157.82, 54.03,70.97, 92.0	158.54, 54.29, 71.19, 91.6
Space group	C2	C2
Resolution (Å)	41.7–1.9 (2.0–1.9)	53–1.8 (1.9–1.8)
Observations	167,473 (24,179)	153,466 (12,563)
Unique reflections	44,765 (6,339)	50,122 (4,473)
I/σ(I)	8.8 (2.7)	11.1 (2.5)
*R*_sym_*^a^*	0.079 (0.444)	0.070 (0.388)
*R*_meas_	0.107 (0.588)	0.085 (0.4750)
*R*_p.i.m_	00.055 (0.297)	0.047 (0.270)
Completeness (%)	94.9 (93.1)	89.2 (54.9)

**Refinement**		
Non-hydrogen atoms	4,075 (including a UDP-MurNAc-Ala-Glu-Lys, 2 Mg^2+^, 1 PO_4_, 1 K, 1 Cl, and 213 waters)	4,408 (including a UDP-MurNAc-Ala-Glu-Lys, and ADP, 2 Mg^2+^, 2 glycerols, and 490 waters)
*R*_cryst_*^b^*	0.196 (0.294)	0.160 (0.258)
Reflections used	42,467 (2,271)	48,057 (1,981)
*R*_free_*^c^*	0.241 (0.337)	0.197 (0.290)
Reflections used	2,984 (138)	2,065 (86)
*R*_cryst_ (all data)*^b^*	0.184	0.161
Average temperature factor (Å^2^)	16	9
r.m.s.d. values from ideal values		
Bonds (Å)	0.015	0.015
Angles (°)	1.7	1.5
DPI*^d^* coordinate error (Å)	0.15	0.12
Ramachandran plot*^e^*		
Favored (%)	97.5	98.0
Outliers (%)	0.4	0.4

*^a^ R*_sym_ = Σ_j_Σ*_h_*|*I_h,j_* − 〈*I_h_*〉|/Σ*_j_*Σ*_h_*〈*I_h_*〉 where *I_h,j_* is the *j*th observation of reflection *h*, and 〈*I_h_*〉 is the mean intensity of that reflection.

*^b^ R*_cryst_ = Σ‖*F*_obs_| − |*F*_calc_‖/Σ|*F*_obs_| where *F*_obs_ and *F*_calc_ are the observed and calculated structure factor amplitudes, respectively.

*^c^ R*_free_ is equivalent to *R*_cryst_ for a 4% subset of reflections not used in the refinement (71).

*^d^* DPI refers to the diffraction component precision index (72).

*^e^* As calculated by MolProbity (30).

##### Analysis of Intracellular Concentrations of Lysine and mDAP

The pool levels of amino acids were determined according to Mengin-Lecreulx *et al.* (44). Exponentially growing cells of *E. coli* BW25113 and *S. aureus* RN4220 were cultivated in 2YT medium (400 ml) at 37 °C. When the *A*_600 nm_ reached 0.75 absorbance units, cultures were rapidly chilled, and cells were harvested in the cold. Extraction of amino acids was performed by using the classical two-step procedure (44): (i) boiling water for 30 min followed by (ii) trichloroacetic acid (TCA, 5% (w/v) final concentration) for 30 min at 0–4 °C. After centrifugation, TCA was extracted from supernatant fractions with ether, and extracts were neutralized and lyophilized. Final solutions were made in 3 ml of water, and the compositions and concentrations of amino acids were determined by injection of aliquots into the Hitachi model 8800 amino acid analyzer.

##### Enzyme Assay

The activity assays of MurE_Sa_ measured the addition of l-Lys to UDP-MurNAc-l-Ala-γ-d-[^14^C]Glu using reaction mixtures (final volume, 50 μl) containing 100 mm Tris-HCl, pH 8.6, 15 mm MgCl_2_, 5 mm ATP, 0.3 mm UDP-MurNAc-l-Ala-γ-d-[^14^C]Glu, l-Lys (varying concentrations), and enzyme (15 μl of an appropriate dilution). The mixtures were incubated for 30 min at 37 °C, and the reaction was terminated by the addition of glacial acetic acid (10 μl) followed by lyophilization. Radioactive substrate and product were then separated by HPLC on a Nucleosil 100C18 5U column (150 × 4.6 mm; Alltech France) using 50 mm sodium phosphate and 7.2 mm sodium hexanephosphonate, pH 2.5/acetonitrile (98.5:1.5, v/v) (18) at a flow rate of 0.6 ml × min^−1^. Radioactivity was detected with a flow detector (model LB506-C1, Berthold) using the Quicksafe Flow 2 scintillator (Zinsser Analytic) at 0.6 ml × min^−1^. Quantification was performed with the Radiostar software.

## RESULTS

### 

#### 

##### Overall Protein Structure of MurE_Sa_

**FIGURE 1.**
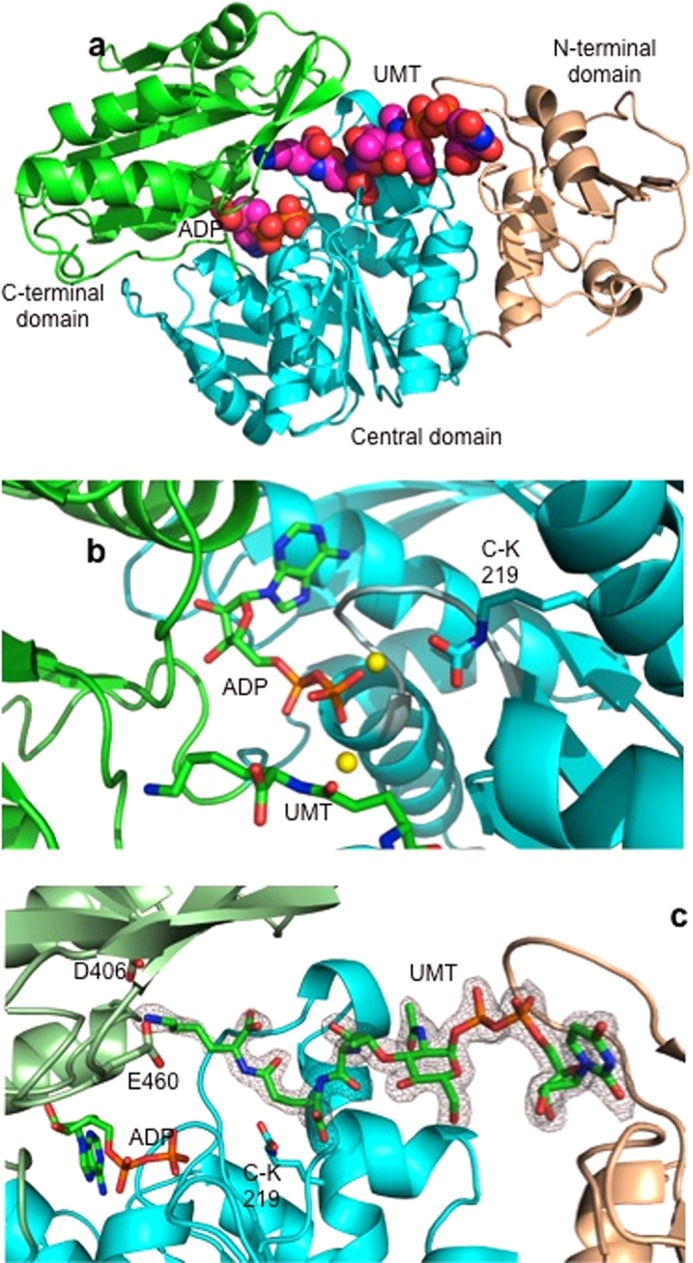
*a*, graphic representation of the overall three-domain structure of MurE_Sa_. The amino-terminal domain (domain 1) is shown in *gold*, the central domain (domain 2) is in *cyan*, and the carboxyl domain (domain 3) is in *green*. ADP and UDP-MurNAc-l-Ala-γ-d-Glu-l-Lys (UMT) are shown in space-filling representation with ADP bound between domains 2 and 3 and the UDP-MurNAc-l-Ala-γ-d-Glu-l-Lys predominantly bound by central domain 2. *b*, close-up representation of the ADP-binding site. ADP is bound within the Walker type-binding site, directly above an α-helix in the region of the TGT*X*GKT consensus sequence shown in *gray*. This sequence forms a discrete loop and amino-terminal end of a helix providing a helix dipole charge positioned immediately adjacent to the α and β phosphorus oxygens of ADP as shown. Magnesium ions are shown as *yellow spheres*, and the position of the carbamylated lysine (*C-K219*) is indicated in relation to the rest of the binding site. The peptide portion of the UMT is shown at the *bottom* of the figure with the l-lysine at the *left-hand end* of the peptide. Loop residues 198–212 have been removed for clarity. *c*, representation of the UMT-binding site and its relationship to the ATP-binding site and associated protein side chain residues. Although most protein-UMT interactions are mediated through the central domain 2 of the protein, those made in relation to the specific l-lysine interactions are derived from amino acid residues in the carboxyl-terminal domain 3. The SIGMAA (73)-weighted 2*F_o_* − Δ*F_c_* electron density using phases from the final model of the half-reduced form is contoured at 2.0 σ level, where σ represents the r.m.s. electron density for the unit cell. Contours more than 1.6 Å from any of the displayed atoms have been removed for clarity. UMT lysine-binding residues Asp-406 and Glu-460 are shown along with carbamylated lysine (*C-K219*). Loop residues 146–156 and 455–475 have been removed for clarity.

We have determined the structure of MurE in complex with UDP-MurNAc-l-Ala-γ-d-Glu-l-Lys both in the presence and in the absence of the cofactor ADP. The structure reveals the enzyme in a ternary product complex with ADP and UDP-MurNAc-l-Ala-γ-d-Glu-l-Lys within the active site in a closed conformation. As seen with other enzymes of this class, MurE_Sa_ is composed of a three-domain, mixed α/β structure. The overall structure, domain architecture, and secondary structure content are very similar to those seen in both the *E. coli* and the *Mycobacterium tuberculosis* enzyme x-ray crystal structures (*Z*-score 21.2, r.m.s.d. 1.48 Å over 454 Cα atoms and *Z*-score 16.2, r.m.s.d. 2.34 Å over 447 Cα atoms, respectively (27, 28, 45)). Domain 1 extends from residues 1 to 98 encompassing the uridine nucleoside-binding site of the UDP-MurNAc-tripeptide product, domain 2 extends from residues 99 to 332 and encompasses most of the rest of the UDP-MurNAc-tripeptide-binding pocket, and finally, domain 3 extends from residues 333 to 493, with the ATP-binding site formed between domains 2 and 3 (Fig. 1*a*). Comparison of the two crystal structures produced in this study reveal no major changes to the overall structure as demonstrated by a measurement of r.m.s.d. of 0.31 Å.

##### ATP-binding Site Structure and Homology

The ATP-binding site in MurE_Sa_ is well defined and is composed of the amino acid elements consistent with those of the nucleotide-binding region of the Mur ligases with a generalized “Walker” ATP-binding motif sequence G*XX*GK(T/S) (46). Within the context of the three MurE ligase enzymes for which there is structural information, this sequence element is extended to TGT*X*GKT, which corresponds to a specific loop, emanating from a parallel section of the 11-bladed central β-sheet structure of the protein, which then runs into an α-helix positioned so as to provide a helix dipole element to the ATP-binding site. This structural feature is conserved in all Mur ligases (Fig. 1*b*).

Although the two structures in this study do not differ substantially, as assessed by comparison of r.m.s.d. values between them, inspection of the ATP-binding site of the product co-crystallization complex shows that two side chains take on a different conformation. These side chains, Phe-300 and Lys-360, are both in the vicinity of the binding site for the adenine ring of ATP. In the case of Phe-300, the phenyl ring is rotated by roughly 90° to accommodate the adenine ring, and this movement displaces a network of two ordered water molecules. The relocation of Lys-360 is more subtle, with the ϵ-amino group still interacting with Asp-356; however, the hydrogen bond has been moved from the OD2 oxygen to the OD1. This movement in effect causes the lysine side chain to move up and out of the binding site. The position of the α- and β-phosphorus atoms of ADP with respect to the helix dipole of the walker motif within the ATP site is replaced by a single phosphate ion in the structure lacking ADP.

As with the *E. coli* and *M. tuberculosis* MurE structures, as well as those of the preceding enzyme in the pathway MurD (47), a specific lysine residue (Lys-219) is post-translationally modified to *N*-carboxylated lysine within the active site region and is thought to be required for positioning of the Mg^2+^-ATP complex. By analogy with MurD, this chemically modified residue orients the γ-phosphate of ATP in such a way that it promotes the generation of a transient UDP-MurNAc-phosphodipeptide intermediate, which then undergoes nucleophilic attack by the incoming l-lysine residue to form the UDP-MurNAc-tripeptide product (Fig. 1*c*).

##### Structural Implications of l-Lysine Selection and mDAP Discrimination

In many Gram-positive organisms, l-lysine is used at the third position of the PG stem peptide, and discrimination against the selection of mDAP is crucial for PG biosynthetic reactions. Such discrimination is accomplished in the presence of both l-lysine and mDAP in the cytoplasm because mDAP is a precursor in the l-lysine biosynthetic pathway (48, 49). Thus, the control of MurE selectivity and stereospecificity is crucial to ensure that the amino acid is selected for entry to the PG pentapeptide third position. The question of how such control is exerted may be addressed by a comparison of the structures of l-lysine- and mDAP-specific MurE enzymes that our results now afford.

**FIGURE 2.**
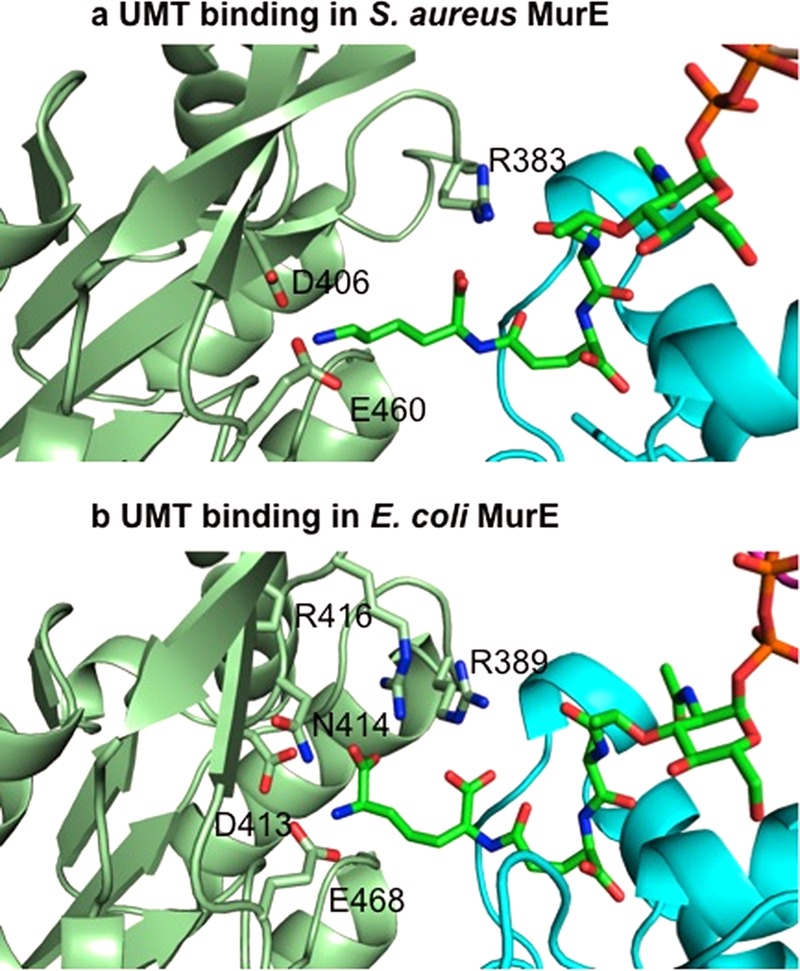
**Active sites of MurE_Sa_ (*a*) and MurE_Ec_ (*b*) in relation to the UDP-MurNAc-tripeptide (product)-binding sites.**

In MurE_Sa_, the l-lysine residue at position three of the UDP-MurNAc-tripeptide ligand is bound by a relatively small number of electrostatic interactions with the protein. The predominant interactions with ϵ-amino group of l-lysine are with the side chain of Glu-460, Asp-406, and the main chain carbonyl group of Ser-456 (Fig. 2*a*). By contrast within the structures of mDAP-dependent *E. coli* and *M. tuberculosis* enzymes, there are four residues with the sequence DNPR (residues 413–416 in *E. coli* and 447–451 in *M. tuberculosis*) first identified by sequence and latterly by structural alignment as being involved in mDAP binding (18, 28), which are principally required for interaction of the mDAP carboxyl group not involved in formation of the stem peptide. Of these residues, the second position Asn and fourth position Arg residues are involved in hydrogen bond interactions with the carboxyl group of mDAP (18, 21). The first Asp residue in this sequence, in concert with a glutamate (Glu-468 in *E. coli*), binds the ϵ-amino group of the l-lysine side chain in UDP-MurNAc-tripeptide (Fig. 2*b*).

Furthermore, inspection and comparison of the structure of the mDAP-specific MurE proteins with that of MurE_Sa_ show that the functionality of this equivalent motif (DNPA in MurE_Sa_) is reduced as only the Asp-406 residue participates in binding in the enzyme-product complex. The other three residues of the proposed motif, in particular Asn-407, are no longer in contact, and the fourth position Arg is completely missing (Fig. 2*a*).

The functionality of Asn-407 appears to be partly replaced by Glu-460, which emanates from a different loop structure connecting β-sheet elements of the protein. The clear role of Arg-416 in the *E. coli* enzyme (Arg-424 in *M. tuberculosis*), for binding of the carboxylate at the d-chiral center of the mDAP substrate, is redundant in MurE_Sa_, and consequently, this residue is replaced by an alanine residue (Ala-409) in this protein, which makes no interaction. This simple substitution would electrostatically discriminate against the selective binding of mDAP to the *S. aureus* enzyme active site.

**FIGURE 3.**
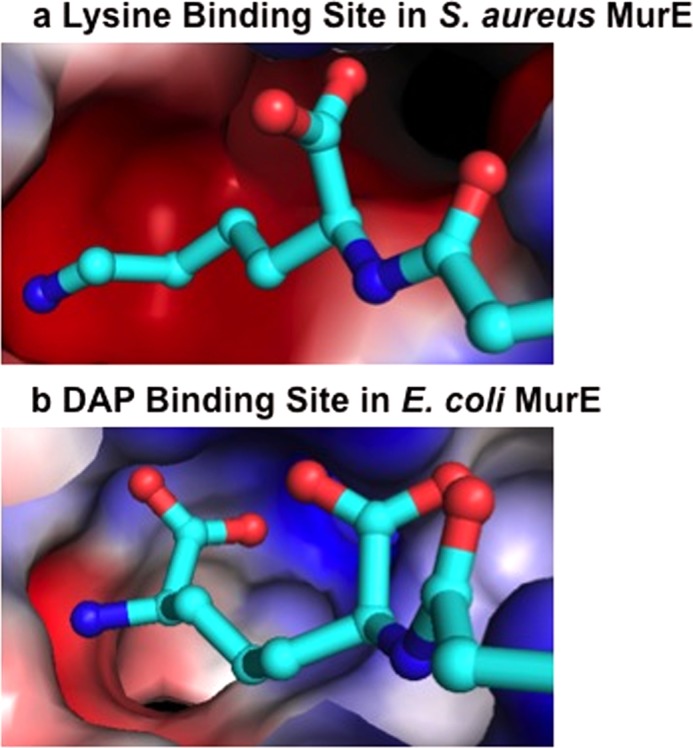
**Electrostatic surface representation of the mDAP- and l-lysine-binding cavities within MurE_Sa_ (*a*) and MurE_Ec_ (*b*) respectively.** The latter cavity is highly acidic, which favors the recognition of the basic l-lysine side chain, whereas that of the *E. coli* enzyme is composed of both basic and acidic residues, favoring the binding of mDAP. The same electrostatic scale is used in each diagram.

Nevertheless, we do not observe an amino acid residue or ordered water molecule, which would be responsible for the absolute positive discrimination for l-lysine in the active site of MurE_Sa_ (19). The selectivity of Mur_Sa_ for lysine may therefore be explained by the following considerations. Firstly, an electrostatic analysis of the active site of l-lysine-specific MurE_Sa_ in comparison with mDAP-specific MurE_Ec_ shows the former to have an overall negative charge that will favor the binding of positively l-lysine side chain (Fig. 3*a*). This would also enable the binding and catalysis of pseudo-substrates such as l-ornithine, which is accepted by MurE_Sa_, but with a 400-fold lower specificity than that with l-Lys (19). By contrast the section of the MurE_Ec_ active site is much less acidic, allowing the binding of the free amino and carboxylate groups of mDAP, the latter of which is also stabilized with a hydrogen bond to Arg-416, as discussed above (Fig. 3*b*). This simple explanation in combination with the observed cytoplasmic concentration ratio of l-lysine to mDAP (see next section) provides a rationale for the discrimination of l-lysine over mDAP and may explain the profound enzymatic specificity observed with respect to these two possible substrates, both of which are present in *S. aureus* by virtue of their *in vivo* biosynthesis as in intermediate (mDAP) or product of the l-lysine biosynthetic pathway.

##### High Levels of Lysine in S. aureus Explain Low Affinity of MurE for its Substrate

As reported previously, the MurE_Sa_ enzyme has an unusually high *K_m_* for l-lysine (0.55 mm) when compared with the *E. coli* DAP-specific MurE (0.04 mm) (19). Therefore, we evaluated the *in vivo* concentrations of these metabolites to establish the ability of MurE_Sa_ to maintain the fidelity of peptidoglycan biosynthesis at the third position in the stem peptide. We measured the levels of DAP and lysine in mid-log cultures of *S. aureus* and *E. coli* as in Ref. 44. As can be seen in Table 2, the intracellular concentration of lysine in *S. aureus* is of the order of 20 mm in this analysis when compared with 0.12 mm for DAP. These results are supported by recent metabolomic and proteomic analysis of *S. aureus* at various stages of growth, which indicate high levels of lysine *in vivo* as the cells move from actively growing to stationary phase (50).

**TABLE 2:** **Pools of DAP and lysine in *E. coli* and *S. aureus***

Amino acid	*E. coli*		*S. aureus*	
	nmol/g of dry wt*^a^*	Pool level*^b^*	nmol/g of dry wt*^a^*	Pool level*^b^*
		*mm*		*mm*
DAP	750	0.4	220	0.12
Lysine	12,300	6.6	36,160	20
Lys/DAP ratio		16.5		166

*^a^* Calculated by assuming a dry weight of 375 mg/liters of culture containing 4.5 × 10^11^ bacteria.

*^b^* Calculated by assuming a cell water content of 1.5 × 10^−15^ liters.

Although MurE_Sa_ has an unusually high *K_m_* value for its amino acid substrate, l-lysine, this should not impose any limitation to cellular growth because the pool level of this substrate is ∼40-fold higher than its *K_m_*, *i.e.* it is saturating with respect to enzyme activity. Our previous kinetic analysis failed to detect any activity of MurE_Sa_ against mDAP (15), whose pool level in any case is in the submillimolar range (19). Thus, although MurE_Sa_ appears to be an inefficient catalyst for its amino acid substrate in comparison with other MurE enzymes in the literature, the structure of its l-lysine-binding site, coupled with the extremely high ratio of lysine to DAP *in vivo*, ensures that l-lysine is inserted into the stem peptide of staphylococcal peptidoglycan.

For clarity we should state that our analysis discriminates neither the lysine isomers (l and d) nor the DAP isomers (*meso* and ll). Although it is reasonable to assume that the d-lysine pool, if any, is low, that of ll-DAP in *S. aureus* may not be negligible because this isomer is the precursor of mDAP in the l-lysine biosynthetic pathway. For instance, the ratio of ll to *meso* isomers of DAP in *E. coli* is approximately one. Therefore, the *S. aureus* mDAP pool is probably lower than the value shown in Table 2.

##### Mutagenesis of the Active Site and Phenotypic Analysis of Mutants

To substantiate the conclusions drawn from the results of the x-ray structure of MurE_Sa_ concerning the l-lysine/mDAP discrimination, site-directed mutagenesis of the DNPA sequence and of the Glu-460 residue crucial for interaction with the ϵ-amino group of the l-lysine substrate was performed. Asp-406, Asn-407, Pro-408, and Glu-460 were replaced by alanine, whereas Ala-409 was replaced by arginine to mimic the *E. coli* sequence (DNPR). Two types of experiments were carried out: (i) examination of the phenotype of *E. coli* cells transformed by plasmids harboring the mutated *murE* genes; and (ii) steady state analysis of the purified MurE mutant proteins.

**FIGURE 4.**
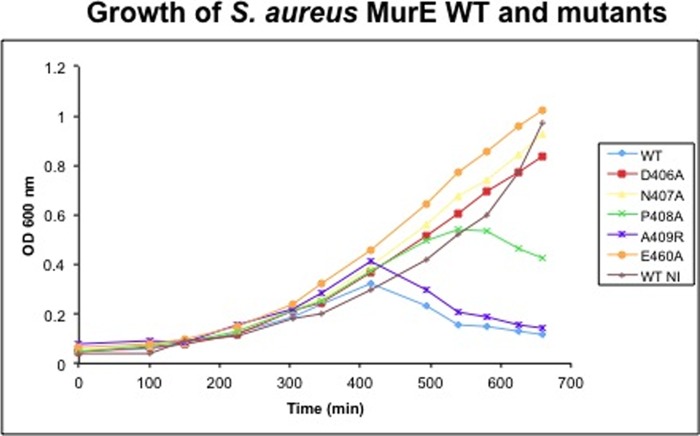
**Effect of wild-type and mutant *murE*_Sa_ overexpression in *E. coli*.** *E. coli* JM83(pREP4, GroESL) cells, transformed by a plasmid harboring either wild-type or mutant *murE*_Sa_ gene, were grown at 22 °C without isopropyl β-d-1-thiogalactopyranoside (*WT NI*) or with 0.5 mm isopropyl β-d-1-thiogalactopyranoside added at *A*_600_ = 0.2 (all others). *OD*, optical density.

The lytic phenotype observed upon overexpression of the wild-type *murE*_Sa_ gene (16) was replicated with A409R and P408A mutants, suggesting that these mutants were endowed with l-lysine-adding activity comparable with the wild type (Fig. 4). Although the growth curve of N407R mutant was similar to that of wild-type *murE*_Sa_, lysis with the P408A mutant occurred with some delay, suggesting a lower *in vivo* activity for this mutant. No lysis could be observed with D406A, N407A, or E460A mutants, suggesting that the *in vivo* activity of the corresponding proteins was nil or very low (Fig. 4).

The mutant proteins were purified as His-tagged forms, and their kinetic parameters were determined (Table 3). The replacement of Asp-406, Asn-407, Pro-408, or Glu-460 by Ala brought about a decrease of affinity for l-lysine as well as of catalytic activity. The extreme cases were mutant protein E460A, whose l-lysine-adding activity was very weak (*K_m_*^l-Lys^ > 20 mm; turnover, 3.2 min^−1^ at 20 mm l-Lys), and N407A, which was totally inactive.

**TABLE 3:** **Kinetic parameters of MurE_Sa_ wild-type and mutant proteins** The concentration of ATP and UDP-MurNAc-l-Ala-d-Glu were fixed at 5 and 0.3 mm, respectively. The concentration of l-lysine varied from 1 to 20 mm. *S. aureus* MurE has no detectable activity against d-lysine when tested at d-lysine concentrations of 20 mm and with enzyme concentrations 500x greater than that used for l-lysine assays. No mDAP-adding activity could be observed for any protein with a 5-μg amount.

Protein	*K_m_*^l-Lys^	*k*_cat_	*k*_cat_/*K_m_*^l-Lys^
	*mm*	*s*^−*1*^	*s*^−*1*^ × *mm*^−*1*^
WT	0.55	4.833	8.787
D406A	2.8	0.001	0.001
N407A	NA*^a^*	NA*^a^*	NA*^a^*
P408A	3.1	0.283	0.091
A409R	>20	—*^b^*	0.003*^b^*
E460A	>20	—*^c^*	0.029*^c^*

*^a^* No detectable activity with 5 μg of protein.

*^b^* The Michaelis-Menten plot was linear up to 20 mm l-lysine. At this concentration, the *k* = *v/E* value was 0.053 s^−1^ (3.2 min^−1^).

*^c^* The Michaelis-Menten plot was linear up to 20 mm l-lysine. At this concentration, the *k* = *v/E* value was 0.58 s^−1^ (35 min^−1^).

Inspection of the MurE_Sa_ structure provides a possible explanation for this in that Asn-407 makes a series of structurally important hydrogen bond interactions within the context of the architecture of the active site region. In particular, it interacts with Glu-460 and Lys-457 as well as the main chain amino group of Ile-464, thereby providing a stabilizing role in that context. These results are consistent with the growth curves; correspondingly, the most active mutant protein (P408A) is the one for which the lytic phenotype is still observed.

The A409R mutant protein had a *K_m_* for l-lysine that was greatly increased as well (*K_m_*>20 mm). Nevertheless, the turnover determined (35 min^−1^ at 20 mm l-lysine) was relatively significant. This was consistent with the observation of a lytic phenotype by the mutant. The A409R mutation was intended to mimic the *E. coli* DNPR sequence and thereby to promote some mDAP-adding activity, of which wild-type MurE_Sa_ is totally devoid (16). However, no mutant had detectable activity with mDAP as the amino acid substrate, eliminating this as a possible contributory factor to the observed growth phenotypes of *E. coli* strains expressing these proteins.

This observation can be reconciled in respect of the crystal structure whereby such a mutation would have a small contribution to neutralizing the overall negative charge of the l-lysine-binding pocket (Fig. 3*a*). Therefore, it appears that the DNPA sequence of MurE_Sa_, despite the limited number of interactions with the amino acid substrate, participates as a whole in the structure and stability of the l-lysine-binding site. Moreover, the fact that the A409R mutation does not promote mDAP incorporation indicates that the DNPA sequence is not the only element responsible for the specificity for the amino acid substrate.

## DISCUSSION

There is considerable interest in the role of MurE in the biosynthesis of peptidoglycan from a number of perspectives. In general, MurE has a central biochemical role in the biosynthetic pathway leading to peptidoglycan and provides the stereospecific selection required for l-lysine or mDAP incorporation. In addition, studies by Gardete *et al.* (51) indicated that disruption or control of expression of the *murE*_Sa_ gene, using an inducible promoter system, has significant transcriptional effects on the *pbp2* and *mecA* genes that encode penicillin-binding proteins 2 and 2A, respectively.

When the *in vivo* level of MurE activity is lowered, an accumulation of the pool of the UDP-MurNAc-dipeptide substrate occurs, which results in a dramatic reduction of methicillin resistance. This accumulated UDP-MurNAc-dipeptide is transferred by MraY to form an undecaprenyl lipid-linked precursor, subverting the normal lipid II-containing pentapeptide route that is used for transglycosylation reactions and incorporated into glycan strands as described in the Introduction (52). These “abnormal” glycan intermediates are not competent for transpeptidation reactions because they lack both the critical third position l-lysine residue of the pentapeptide stem, to which a pentaglycyl chain is attached via the FemXAB ligase system (20), and fourth and fifth position d-alanine residues that are required for cross-linking reactions yielding the mature PG layer. Thus, inhibition of MurE may have clinical value not only from a direct antimicrobial perspective but also as a method of sensitizing otherwise resistant *S. aureus* to existing and new generation β-lactam compounds.

Moreover, the above is also linked to observations upon the disruption of the mDAP decarboxylase gene: LysA, the penultimate enzyme in the bacterial pathway for l-lysine synthesis. Mutants of *S. aureus* LysA are reported to be linked with a decrease in virulence in a murine bacteremia model of infection (53, 54) and a decrease in the minimum inhibitory concentration for methicillin of over 100-fold in the highly methicillin resistance COL strain of *S. aureus* (55).

In this study, we report for the first time the molecular interactions required for l-lysine selection over mDAP in any Gram-positive bacterial species. Surprisingly, we observe that there is a lack of specific interactions mediated by amino acids in the active site of MurE_Sa_ in favor of an overall electrostatic contribution from the active site cleft that favors binding of the positively charged l-lysine substrate side chain. This lack of specificity is no doubt the origin of the relatively poor affinity of MurE_Sa_ for its amino acid substrate in comparison with DAP specific enzymes (19). The additional d-carboxyl group present in the side chain of mDAP would be electrostatically repelled in the MurE_Sa_ active site, which in addition to the low, *in vivo* concentration of mDAP relative to l-lysine in the cytoplasm leads to overall exclusive incorporation of l-lysine into the peptidoglycan stem peptide.

A mutational analysis of the enzyme provides further experimental evidence for these relationships but also suggests differences in conformation between the product complex observed in the structure and the transition state. This is consistent with the notion of domain movements within the Mur ligases in general during catalysis as described previously (56) and has been studied most extensively with MurD (57, 58).

A recent metabolomics and proteomic study of *S. aureus* cells under glucose starvation conditions revealed a striking intracellular accumulation of l-lysine, used in these experiments even in the presence of a functional l-lysine biosynthetic pathway, where the constituent enzyme showed little variation in abundance during the growth of *S. aureus* (50). This analysis was confirmed by our own investigations, which indicate very high levels of lysine when compared with DAP in the cytoplasm.

Thus, even moderate inhibition of MurE_Sa_ activity via novel inhibitory molecules could have profound antimicrobial activity, especially when used in combination with existing inhibitors, as is clear from Ref. 50. Taking advantage of the perturbation of flux through the peptidoglycan synthetic pathway by specifically targeting the comparatively poor but specific kinetic properties of MurE_Sa_ using inhibitors of the lysine biosynthetic pathway would reduce the overall concentration of l-lysine *in vivo*, rendering *S. aureus* more sensitive to drugs targeting the MurE_Sa_ l-lysine-binding site. Additionally, targeting MurE_Sa_ could reduce the number of sites within the *S. aureus* peptidoglycan available for transpeptidation and therefore resensitize methicillin-resistant *S. aureus* (MRSA) to β-lactam therapy (*e.g.* Ref. 50).

In a wider consideration of lysine in the context of *S. aureus*, we note that the membrane protein MprF modifies bacterial membrane lipids using lysyl-tRNA^Lys^ as a substrate, which leads to electrostatic repulsion of the membrane-damaging peptides (59–61), and that the lysine biosynthetic pathway has long been considered to be a viable antimicrobial target (48, 49). Thus, strategies aimed at altering lysine metabolism in *S. aureus* may have multiple antimicrobial effects especially when used synergistically with existing drugs, potentiating the effect of β-lactams against methicillin-resistant *S. aureus* as has been recently demonstrated (62, 63).

A large number of research groups have pursued a general approach to the inhibition of Mur ligase and pathway-related amino acid ligase enzymes in the past (64–69), but this study has now provided evidence for a more focused approach based upon the properties of MurE_Sa_. Whether these properties extend to other l-lysine-specific enzymes, which are found in many Gram-positive pathogens, is currently being explored in this laboratory.

Finally, the dual targeting strategy of disabling both a Mur ligase activity and the supply of its cognate amino acid discussed here for MurE_Sa_ could also be envisaged for targeting MurD and MurF in which in both of these cases, antimicrobials have been developed that target d-glutamate production and d-alanyl-d-alanine production (70).

## Footnotes

4 The abbreviations used are:

PG: peptidoglycan
DAP: diaminopimelic acid
mDAP: *meso*-diaminopimelic acid
UDP-MurNAc: UDP-*N*-acetylmuramic acid
MurE: UDP-*N*-acetylmuramoyl-l-alanyl-d-glutamate:meso-2,6-diaminopimelate ligase
PBP: penicillin-binding protein
UMT: UDP-MurNAc-l-Ala-γ-d-Glu-l-Lys
Bicine: *N*,*N*-bis(2-hydroxyethyl)glycine
AMP-PNP: adenosine 5′-(β,γ-imido)triphosphate
r.m.s.d.: root mean square deviation.
